# Supplementary material for: Comparative genomics provides new insights into the diversity, physiology, and sexuality of the only industrially exploited tremellomycete: Phaffia rhodozyma
Source: BMC Genomics. 2016 Nov 9;17:901. doi: 10.1186/s12864-016-3244-7 (PMC5103461; doi:10.1186/s12864-016-3244-7)
Supplement: Additional file 6: — List of orphan genes with links to PFAM (related to Additional file 1: Table S1). (ZIP 1428 kb) [file 12864_2016_3244_MOESM6_ESM.zip › PFAM_UNIQUE/G01076_P.html]

#                                                                            --- full sequence --- -------------- this domain -------------   hmm coord   ali coord   env coord
  
# target name        accession   tlen query name           accession   qlen   E-value  score  bias   #  of  c-Evalue  i-Evalue  score  bias  from    to  from    to  from    to  acc description of target
  
#------------------- ---------- ----- -------------------- ---------- ----- --------- ------ ----- --- --- --------- --------- ------ ----- ----- ----- ----- ----- ----- ----- ---- ---------------------
  
G01076\_P             -            692 FAM75                PF14650.1    373       1.3    7.0   4.7   1   1   0.00083       2.5    6.1   4.7    75   126    79   129    45   163 0.76 - FAM75 family  
G01076\_P             -            692 Gemini\_AL1\_M         PF08283.6    106      0.06   11.9   0.0   1   2      0.25   1.5e+03   -2.3   0.0    68    84   409   425   403   436 0.72 - Geminivirus rep protein central domain  
G01076\_P             -            692 Gemini\_AL1\_M         PF08283.6    106      0.06   11.9   0.0   2   2   6.5e-05      0.39    9.3   0.0    49    94   511   557   505   562 0.86 - Geminivirus rep protein central domain
